# LHRH-Conjugated Drugs as Targeted Therapeutic Agents for the Specific Targeting and Localized Treatment of Triple Negative Breast Cancer

**DOI:** 10.1038/s41598-020-64979-1

**Published:** 2020-05-19

**Authors:** J. D. Obayemi, A. A. Salifu, S. C. Eluu, V. O. Uzonwanne, S. M. Jusu, C. C. Nwazojie, C. E. Onyekanne, O. Ojelabi, L. Payne, C. M. Moore, J. A. King, W. O. Soboyejo

**Affiliations:** 1 Department of Mechanical Engineering, Higgins Lab, 100 Institute Road, Worcester Polytechnic Institute (WPI), Worcester, MA 01609 USA; 2 Department of Biomedical Engineering, Gateway Park Life Sciences Center, 60 Prescott Street, Worcester Polytechnic Institute (WPI), Worcester, MA 01605 USA; 3 Department of Pharmaceutical Microbiology and Biotechnology, Nnamdi Azikiwe University, 420110 Ifite Awka, Anambra State Nigeria; 4 Department of Material Science, African University of Science and Technology, Km 10 Airport Road, Abuja, Nigeria; 5 RNA Therapeutics Institute, University of Massachusetts Medical School, 368 Plantation Street, Worcester, MA 01605 USA; 6 Department of Psychiatry, Center for Comparative NeuroImaging, University of Massachusetts Medical School, 303 Belmont Street, Worcester, MA 01604 USA; 7 Department of Biology & Biotechnology, Gateway Park Life Sciences Center, 60 Prescott Street (Gateway Park I), Worcester Polytechnic Institute (WPI), Worcester, MA 01605 USA

**Keywords:** Breast cancer, Drug delivery

## Abstract

Bulk chemotherapy and drug release strategies for cancer treatment have been associated with lack of specificity and high drug concentrations that often result in toxic side effects. This work presents the results of an experimental study of cancer drugs (prodigiosin or paclitaxel) conjugated to Luteinizing Hormone-Releasing Hormone (LHRH) for the specific targeting and treatment of triple negative breast cancer (TNBC). Injections of LHRH-conjugated drugs (LHRH-prodigiosin or LHRH-paclitaxel) into groups of 4-week-old athymic female nude mice (induced with subcutaneous triple negative xenograft breast tumors) were found to specifically target, eliminate or shrink tumors at early, mid and late stages without any apparent cytotoxicity, as revealed by *in vivo* toxicity and *ex vivo* histopathological tests. Our results show that overexpressed LHRH receptors serve as binding sites on the breast cancer cells/tumor and the LHRH-conjugated drugs inhibited the growth of breast cells/tumor in *in vitro* and *in vivo* experiments. The inhibitions are attributed to the respective adhesive interactions between LHRH molecular recognition units on the prodigiosin (PGS) and paclitaxel (PTX) drugs and overexpressed LHRH receptors on the breast cancer cells and tumors. The implications of the results are discussed for the development of ligand-conjugated drugs for the specific targeting and treatment of TNBC.

## Introduction

Breast cancer is the most commonly diagnosed cancer and the second most frequent cause of death in women^1^. In general, breast tumors are intrinsically heterogeneous in nature, making them difficult to detect and treat^2^. Approximately, 75–80% of breast cancers are hormone receptor-positive^2,3^. Also, these overexpressed receptors are usually estrogen and/or progesterone receptors^2,3^. However, Triple Negative Breast Cancer (TNBC) (which represents approximately 10–17% of all breast cancers) does not express estrogen receptors (ER), or progesterone receptors (PR), or the human epidermal growth factor receptor 2 gene (HER2)^4–8^. In addition, TNBCs also exhibit distinctive clinical features^7,8^ and are more common in younger patients^6^ and African American/African women^9^.

TNBC is an aggressive and immunopathology subtype of breast cancer that usually does not respond to drugs that target ER, PR and HER2^6^. Furthermore, since the most common and conventional breast cancer diagnosis and treatment techniques tend to focus and target ER, PR and HER2, it is often difficult to detect^10^ and treat^11^ TNBCs with conventional targeted hormonal therapy and chemotherapy. The challenges associated with TNBCs result in relatively poor prognoses, accentuated side effects, aggressive tumor growth, and limited targeted therapies^11^. Other common therapeutic approaches, such as bulk chemotherapy and radiation therapy, lack specificity and are associated with severe side effects^12^.

Recent efforts indicate that breast cancer cells can exhibit or acquire intrinsic resistance to chemotherapeutic drugs^13^. Such drug resistance is often associated with complicated tumor microenvironments^13^. Furthermore, in the case of bulk chemotherapy, only very small fractions of the drugs reach the tumor sites of interest^14^. This results in side effects^15^ that are associated with drug interactions with non-tumor-bearing healthy tissue and organs. In most cases, targeted cancer drug delivery systems can attach specifically to antibodies, peptides and hormonal receptors that have been developed for the treatment of tumors that overexpress these receptors^16–20^. Typical examples include: HER2, PR and ER receptors^3^. However, TNBC presents challenges since it is not well targeted by conventional cancer drugs. There is, therefore, a need to develop chemotherapeutic drugs for the effective targeting and treatment of TNBC.

Prior work has shown that LHRH receptors are expressed in over 50% of human breast cancer specimens obtained from a non-selected patient cohort characterized by TNBC^21,22^ as well as in MDA-MB-231 cell lines^11,20^. Dharap *et al*.^23^ have also reported that LHRH receptors are overexpressed in human breast cancer, ovarian cancer, as well as prostate cancer cells that are below the detection limits of PCR in normal human organs (lung, liver, kidneys, spleen, muscle, heart, thymus). In another study, LHRH was conjugated to a lytic peptide, hecate, and a 15-amino acid segment of the β-chain of chorionic gonadotropin^24^ and the resulting drug-conjugates were used to treat human prostate xenografts in nude mice^24^. Although, significant work has been carried to explore the overexpression of LHRH receptors in many types of cancer^11,12,15,20–22,25^, some studies have shown that the binding affinity of the receptor in prostate and in other tumor-based tissues might be lower than those found in the pituitary^26–31^. In our work, we only focus on targeting triple negative breast cancer cells that have been shown to overexpressed LHRH receptors^11,12,15,20,21,24^.

Other efforts have explored cytotoxic analogs of various peptides containing doxorubicin with AEZS-108 (also known as AN-152) that consist of doxorubicin linked to the LHRH agonist, [D-Lys6] LHRH^21,32^. A pilot study^33^ has also used immunohistochemistry, RT-PCR, and Western blot analysis to reveal that LHRH receptors are expressed on TNBC tissues. However, to the best of our knowledge, there have been no prior efforts to conjugate bacterially synthesized prodigiosin cancer drug (a secondary metabolite and tripyrrole red pigment) to LHRH (molecular recognition unit) that can improve drug specificity in the targeting of TNBC.

In this paper, LHRH-conjugated prodigiosin and LHRH-conjugated paclitaxel were studied as model cancer drugs. These were synthesized by conjugating [D-Lys6]LHRH to prodigiosin and paclitaxel at the epsilon (ε) amino side chain of the D-Lys6 moiety at position 6 of the [D-Lys^6^]LH-RH (pyroGlu–His–Trp–Ser–Tyr–d-Lys–Leu–Arg–Pro–Gly–NH_2_). The conjugation was successfully accomplished without the loss of the drugs’ abilities to bind to LHRH receptors^34^. The structures produced by the conjugation reactions are characterized using Fourier Transform Infra-Red (FTIR) spectroscopy and Liquid Chromatography–Mass Spectrometry (LC-MS). The effects of the LHRH-conjugated prodigiosin and paclitaxel drugs (on cancer cells/tissue) are then elucidated under *in vitro* (experiments with MDA-MB-231 TNBC cell line) and *in vivo* conditions (using an athymic nude mouse model induced with TNBC xenograft tumors). The conjugated LHRH-prodigiosin and LHRH-paclitaxel are shown to specifically target and eliminate or shrink TNBC xenograft tumors at early, mid and late stages of breast tumor progression. The implications of the results are then discussed for the specific targeting and localized treatment of TNBC.

## Results and Discussion

### Drug conjugation and characterization

Prodigiosin (PGS) which is also known as 4-methoxy-5-[(*Z*)-(5-methyl-4-pentyl-2*H*-pyrrol-2-ylidene)methyl]-1*H*,1′*H*-2,2′-bipyrrole contains a C-6 methoxy substituent in the 4-methoxy-2,20-bipyrrolyl ring. The purity of the prodigiosin that was synthesized for conjugation to LHRH was characterized to be 92.5%^35,36^. The presence of the hydrophilic linker (NHS) creates sites for reactions with the methoxy group that is present in the prodigiosin molecule^37^. The methoxy group (–OCH_3_) on the PGS also has a high electron density and exhibit a tendency to attack the nucleophilic center of the carbonyl group that is present in the NHS linker.

With the presence of EDC, the high electron density attacks the PGS linkages, causing the electrostatic cleavage of the proton from the N–H group, thus linking the LHRH. The reaction with the secondary amine (NH) creates stable amide linkages that do not easily break down. Thus, in the presence of the LHRH molecules, NHS ester crosslinks or couples to the ε-amines to the lysine side chains, and to the α-amines in the N-terminals.

In the case of paclitaxel (PTX), the native lysine ε-amines groups of the LHRH-peptide were targeted for the drug coupling (See Eqs. 1 and 2).

1 $$OH-2{\prime} -PTX+Succinic\,Anhydride\to PTX-2{\prime} -{O}_{2}PTX\,{O}_{2}\,OCC{H}_{2}\,C{H}_{2}\,C{O}_{2}\,H(PTXSCT)$$

2 $$LHRH-N{H}_{2}\,+PTXSCT\to \frac{NHS/EEDG}{DMF}\to LHRH-NH-PTX(PTX-LHRH)$$

The targeting moieties were attached to PTX via the 2-hydroxyl group (on one of its side chains) in the presence of heterobifunctional linkers. The major function of these linkers is to hold the segment of the drug and LHRH peptide together sufficiently enough for the ligands to be attached specifically to the target receptors on the cancer cells/tumor^38^.

**Figure 1:**
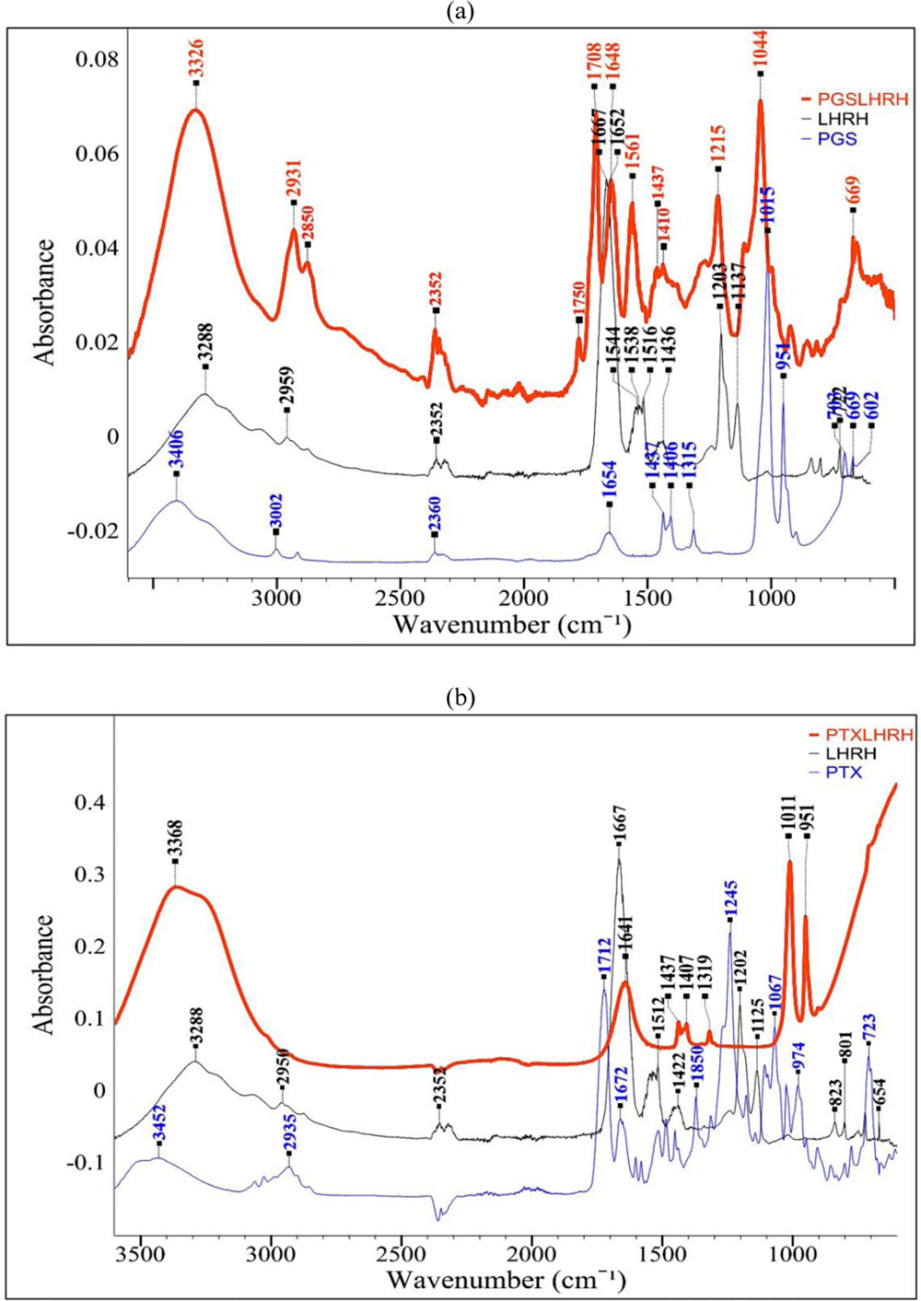
FTIR spectra showing: (**a**) LHRH-conjugated PGS drug and (**b**) LHRH-conjugated PTX drug.

**Figure 2:**
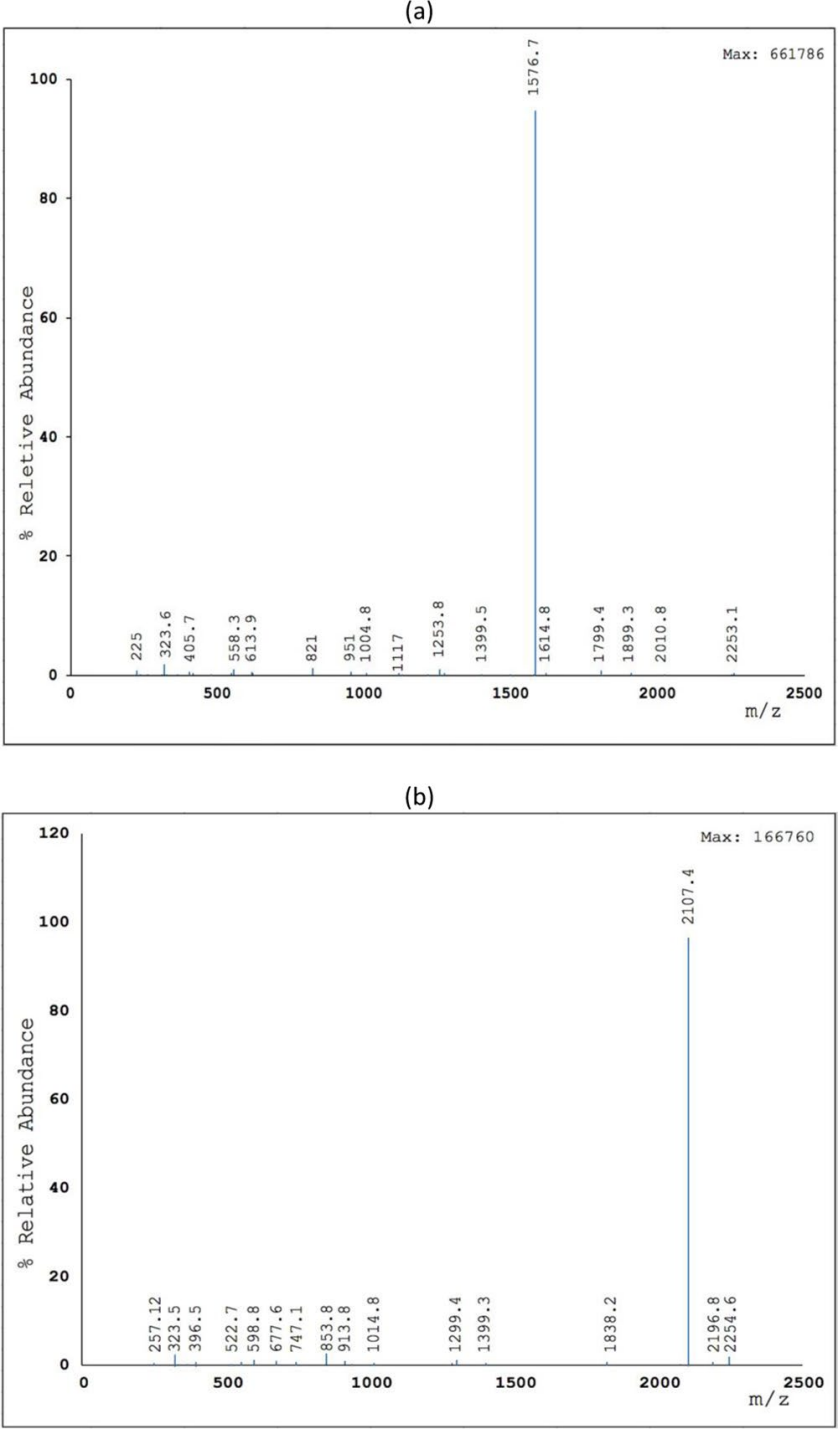
LC-MS spectra of (**a**) PGS-LHRH drug and (**b**) PTX-LHRH drug.

The LHRH-conjugation to prodigiosin was confirmed using a combination of FTIR (Fig. 1a,b) and LC-MS spectra (Fig. 2a,b). The FTIR spectral analysis of LHRH peptide revealed the presence of characteristic amine bands of –NH (~1545 cm^−1^), which disappear after conjugation to PGS and PTX. The spectra from Fig. 1 clearly shows the formation of the amide bond. The increased intensity of the C=O band (Amide/peptide bond) at 1648 for LHRH-conjugated PGS confirm the bonding and conjugation of PGS to LHRH (Fig. 1a). In both cases (Fig. 1a,b), the LHRH-conjugated drugs exhibited typical amide (covalent or peptide) bond signatures at around 1648 cm^−1^ and 1641 cm^−1^ for PGSLHRH and PTXLHRH, respectively.

Finally, the LC-MS spectra exhibited a molecular ion (m/z) peak of pigment that corresponds to the mass-to-charge ratio of PGS-LHRH and PTX-LHRH with their respective molecular weights. In general, the LC-MS results are evidence that LHRH-conjugated PGS and LHRH-conjugated PTX were formed during the conjugation process.

### LHRH Receptors staining, siRNA knockdown, RT-qPCR quantification, *In vitro* cell viability and drug uptake

**Figure 3:**
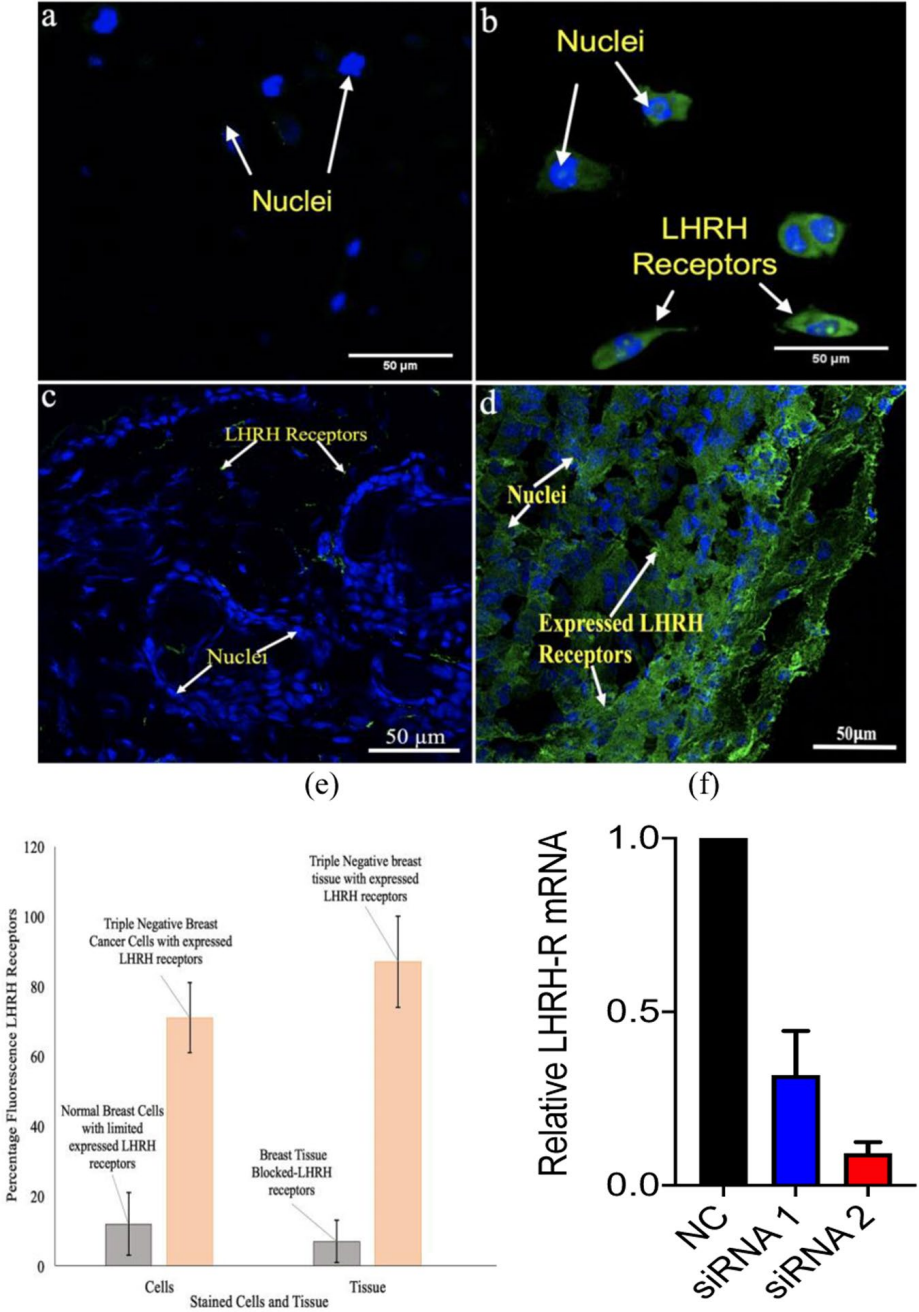
Confocal fluorescence images showing the expression of LHRH receptors (green stains) of (**a**) non-tumorigenic epithelial breast cell line (MCF 10 A) (**b**) Triple negative breast cancer cells (MDA-MB 231) (c) Blocked LHRH antibody receptors on triple negative breast tissue (**d**) Stained LHRH triple negative breast tissue at 40 x magnification (**e**) Quantified fluorescence LHRH receptors in cells and tissue of TNBC. (**f**) Detection of LHRH-R knockdown by RT-qPCR.

Results in Fig. 3a,b show expression of LHRH receptors (green stain) on non-tumorigenic epithelial breast cell line (MCF 10 A) compared to those of triple negative breast cancer cells (MDA MB 231) via immunofluorescence staining. Results showed that evidence of LHRH receptors on TNBC.

In a similar fashion, LHRH receptors are seen to be overexpressed on unblocked LHRH antibody receptors stained TNBC tissue. In the case of blocked LHRH TNBC cells, the receptor expression obtain from fluorescence confocal microscope was very low (Fig. 3c) as compared to those that were unblocked (Fig. 3d). In both cases (Fig. 3a-d), we quantify the percentage fluorescence LHRH receptors as shown in Fig. 3e. These results provide evidence of expression of LHRH receptors on TNBC. Furthermore, results from our knock down experiment using two sets of siRNA show that we knocked down the LHRH receptor in MDA-MB-231 cells and observed a ~70% and 90% reduction of LHRH receptor transcript levels (Fig. 3f). Clearly, knockdown of LHRH receptor significantly reduces the enhanced delivery of PGS and PTX achieved by LHRH peptide conjugation.

**Figure 4:**
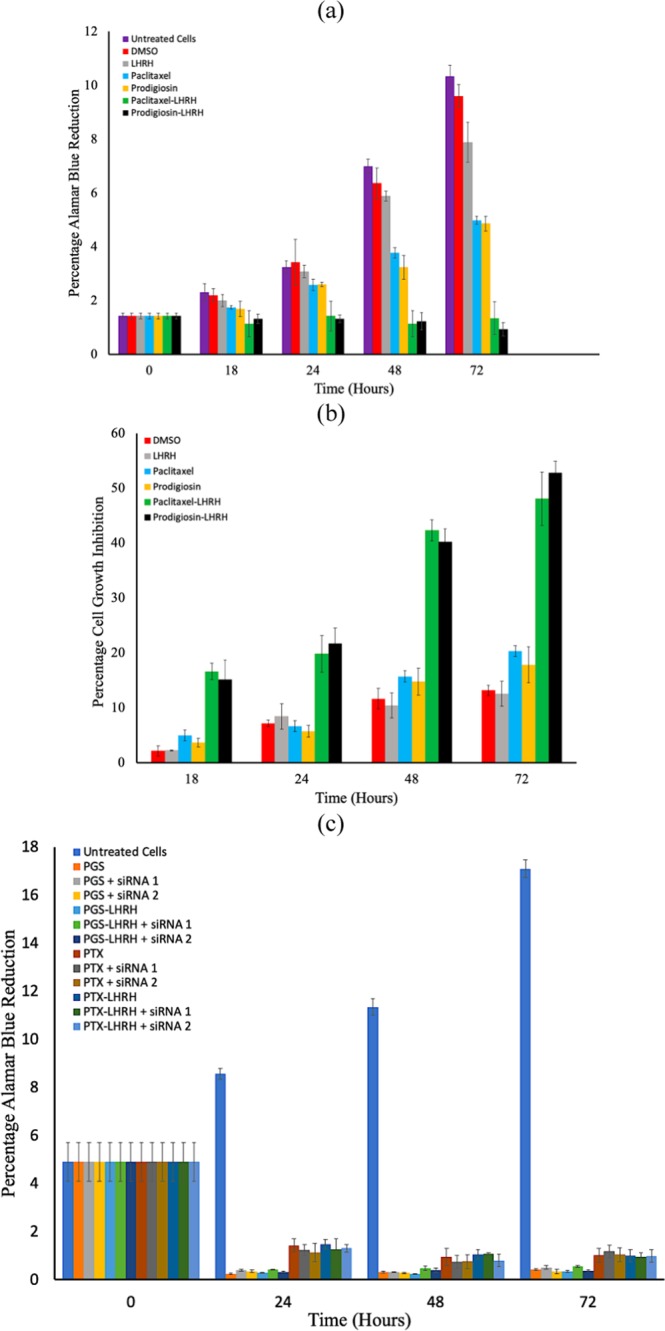
(**a**) Percentage alamar blue reduction for breast cancer breast cells (**b**) Percentage cell growth inhibition (**c**) Percentage alamar blue reduction for knocked down LHRH receptors of breast cancer cells (10^4^ cells/well) co-incubated with 5 µM of DMSO, LHRH, paclitaxel, prodigiosin, LHRH-conjugated PTX and LHRH-conjugated PGS drugs for the period of 72 h. The data presented are the average of three independent experiments. (in both cases n  =  3, p  <  0.05).

Figure 4a compares the viability of untreated breast cancer cells with those treated with drugs after 18, 24, 48 and 72 h of post-treatment. For all of the durations and concentrations considered, the cell viability was slightly lower for PGS-LHRH than for PTX-LHRH only at day 72. Among the cells exposed to 5 µM of LHRH, PGS, PTX, PGS-LHRH, PTX-LHRH drugs or DMSO control, the conjugated drugs (PGS-LHRH and PTX-LHRH) had a greater effect on cell growth, as shown by the lower percentage of alamar blue reduction values. A similar trend was also explored when cells were exposed to increasing drug concentration. Consequently, by isolating the effect of DMSO alone (DMSO is the solvent used to dissolve the drugs), it was observed that there was no significant effect of DMSO on cell viability, when compared to that of LHRH-conjugated drugs. Therefore, the assay revealed that LHRH-conjugated PGS or LHRH-conjugated PTX were more specific than PGS, PTX or LHRH in their targeting of breast cancer cells.

The results presented in Fig. 4b show clearly that LHRH-conjugated PGS was more effective in inhibiting the growth of MDA-MB-231 cells than the LHRH-conjugated PTX. This is because increased inhibition also implies higher cytotoxicity due to drug-treatment. Therefore, at cellular level, both the LHRH conjugated PGS LHRH-conjugated PTX exhibited higher levels of inhibition than those of PGS, PTX or LHRH alone. This implies that, due to the specificity of PGS-LHRH or PTX-LHRH, it creates more cytotoxicity to triple negative breast cancer cells than LHRH, PTX or PGS alone. This makes the PGS-LHRH or PTX-LHRH more active in reducing TNBC cell viability than LHRH, PTX or PGS.

In the presence of the siRNA as shown in Fig. 4c, the cell viability, expressed as the percentage alamar blue reduction, decreased after 24, 48 and 72 hours in a similar fashion of treatment with 5 µM PGS, PTX, PGS-LHRH and PTX-LHRH. Clearly, at times 24, 48 and 72 hours, there were no significant differences in cell viability between PGS and LHRH-conjugated PGS when the cells were treated with the siRNAs. Similar observations were made between PTX and LHRH-conjugated PTX when the cells were treated with the siRNAs. Consequently, the unconjugated and LHRH-conjugated drugs exhibited similar anti-proliferative effects on the cells due to the suppression of LHRH receptor-mediated drug entry into the cells. Without cell treatment with siRNA, the results in Fig. 4(a) showed that the LHRH-conjugated drugs significantly reduced cell viability than the unconjugated drugs due to the specific targeting of the cells. This result is attributed to the specific interactions between the LHRH and the LHRH receptors in the absence of the knock down, and the reduced access of the conjugated or conjugated drugs after the knock down of the cell LHRH receptors by the siRNA.

**Figure 5:**
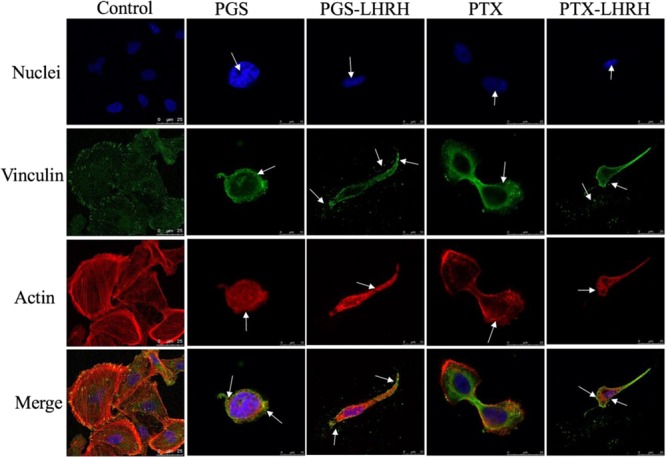
Confocal fluorescence images showing cellular uptake and cytotoxicity comparison of MDA-MB-231 cells 6 hours after their incubation with 30 μM of PGS, PGS-LHRH, PTX or PTX-LHRH (arrows indicate the structural changes in the nuclei structure (blue), actin cytoskeleton structure (red) and vinculin structure (green).

Furthermore, from the confocal fluorescence images of drug-interacted cells (Fig. 5), it is clear that treatment with the drugs result in the degradation, disorganization and depolymerization of the actin filaments and vinculin structures. The drugs also disrupted the cancer cell membranes and cytoskeletal actin structures. These disruption and disintegration give rise to apoptosis and cell death^39–42^. This phenomenon was more evident in LHRH conjugated drugs (PGS-LHRH and PTX-LHRH) than unconjugated drugs (PGS and PTX). In general, the current results show that the conjugation of the cancer drugs to the LHRH peptide increases the selectivity, effectiveness, and uptake of anticancer drugs to TNBC, due to the presence of overexpressed LHRH receptors on the surfaces of the TNBC^20,43,44^.

### *In vivo* tumor development, shrinkage and elimination

**Figure 6:**
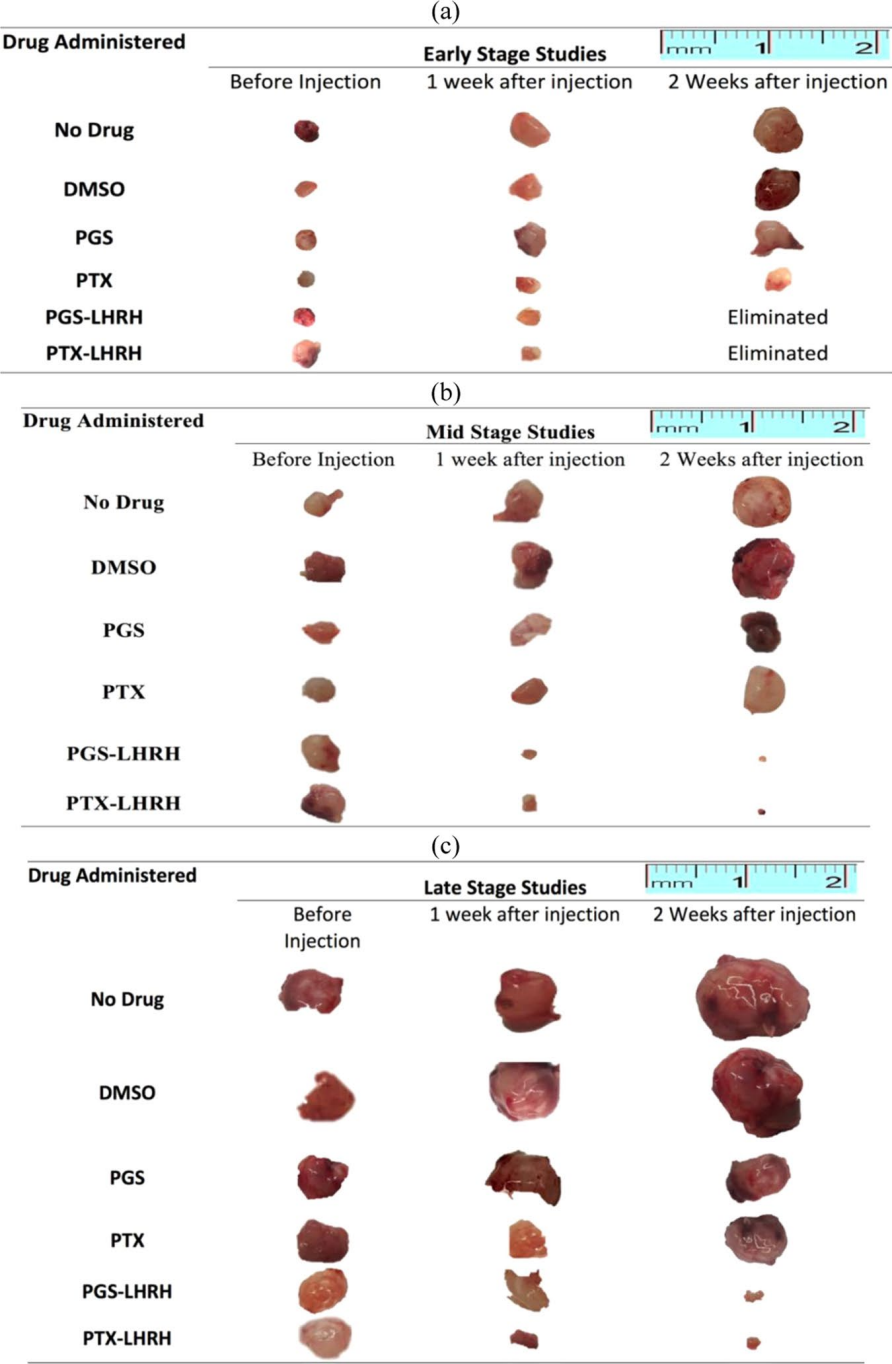
Representative tumor images of induced subcutaneous triple negative breast xenografts tumor treated with two IV injections of PGS-LHRH, PTX-LHRH, PGS, PTX and DMSO for (a) Day-14 (b) Day -21 and (c) Day – 28 treatment groups, respectively.

**Figure 7:**
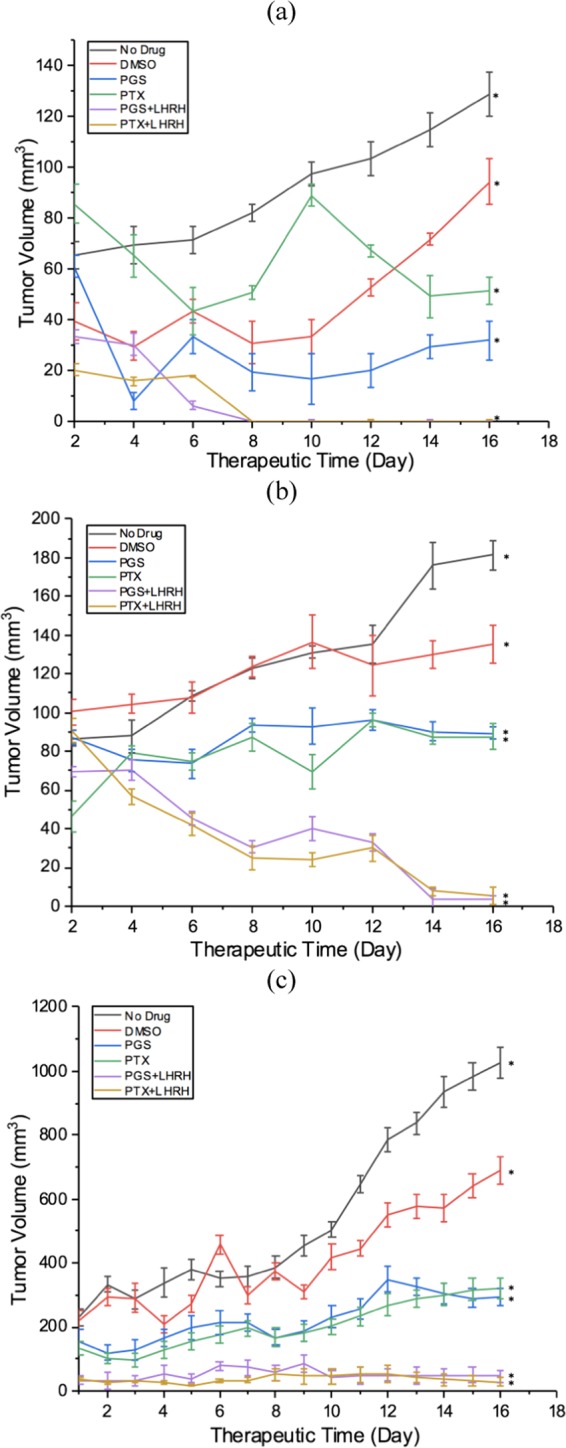
Anti-tumor activity and tumor shrinkages of induced subcutaneous xenografts tumor on female athymic nude mice treated with two IV injections of PGS-LHRH, PTX-LHRH, PGS, PTX and DMSO for (**a**) 14-day study (**b**) 21-day study (**c**) 28-day study (n = 3, ^*^p < 0.05 vs. control).

The mean tumor volumes observed in the mice before treatment on day 14, day 21 and day 28 were ~67 ± 11 mm^3^, 98 ± 29 mm^3^ and 230 ± 18 mm^3^, respectively. In the case of the day-14 treatment group, the tumors were completely eliminated from the mice two weeks after the injection of 10 mg/kg of LHRH-conjugated PGS or LHRH-conjugated PTX into each mouse (one dose per week) (Figs. 6a and 7a). In contrast, the unconjugated PGS and PTX drugs resulted in some tumor shrinkage and final tumor sizes of ~ 29.42 mm^3^ (PGS) and 49.1 mm^3^ (PTX), respectively (Fig. 7a).

In the case of the 21-day group treatment, significant tumor shrinkage was observed after about two weeks (one per week) of administration of 10 mg/kg of LHRH-conjugated PGS and LHRH-conjugated PTX to each mouse, when compared to the tumor shrinkage associated with PGS and PTX. The resulting tumor volumes observed after treatment with the PGS-LHRH and PTX-LHRH were 3.93 mm^3^ and 7.76 mm^3^, respectively (Figs. 6b and 7b). These are much smaller than the tumor volumes of mice that were treated with the non-conjugated PGS and PTX, which resulted in tumor volumes of 90.11 mm^3^ and 86.83 mm^3^, respectively. This implies that, for the 21-day treatment group, there was ~ 95.6% decrease in the xenograft tumor volume after the administration of LHRH-conjugated PGS, compared to that associated with unconjugated PGS. Similarly, a 91% decrease in xenograft tumor volume was observed when PTX-LHRH was used instead of PTX (Fig. 7b).

Finally, in the case of the 28-day treatment group, significant tumor shrinkage was also observed in the xenograft tumors (49.9 mm^3^ and 29.4 mm^3^ for PGS-LHRH and PTX-LHRH, respectively), during the two weeks of drug administration. However, in the case of the mice treated with unconjugated PGS and PTX drugs, the resulting average tumor volumes were 300.3 mm^3^ and 299.2 mm^3^, respectively (Figs. 6c and 7c).

The percentage reduction in xenograft tumor volume for LHRH-conjugated PGS and LHRH-conjugated PTX was 83.4% and 90.2%, respectively, as compared to the unconjugated PGS and PTX drugs (Figs. 6c and 7c). The above results show clearly that, in each of the treatment groups (14-day, 21-day and 28-day), LHRH-conjugated PGS and LHRH-conjugated PTX were more effective in shrinking or eliminating the tumors than PGS and PTX alone.

### *Ex vivo* adhesion of cancer drugs to breast tumors and immunofluorescence staining

**Figure 8:**
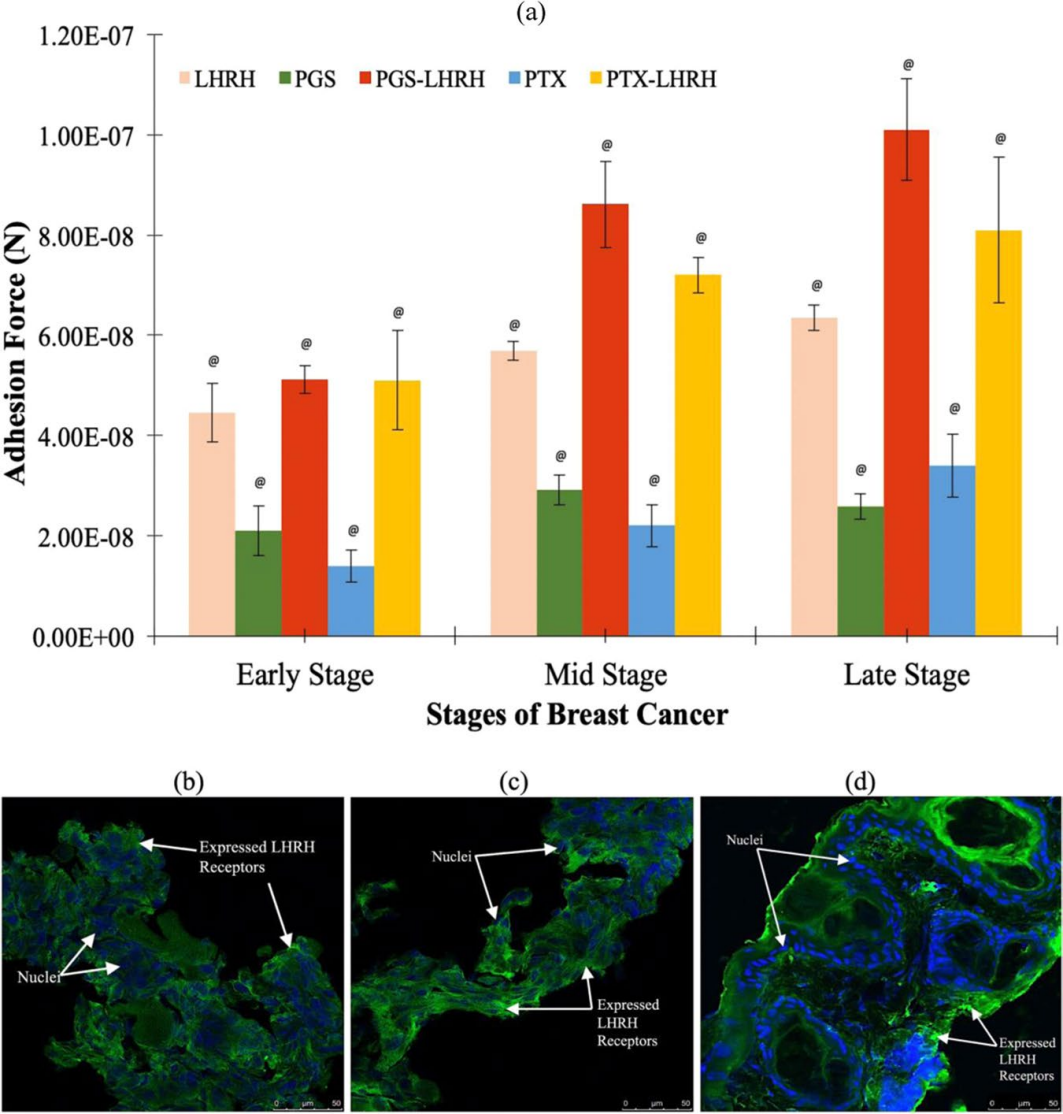
(**a**) Summary of measured pull-off force/adhesion forces between drug-coated AFM tip and early stage, mid stage and late stage triple negative breast tumor (n = 5, ^@^p < 0.001 vs. control). Immunofluorescence staining of expressed LHRH receptors on (b) early stage (c) mid stage (d) late stage triple negative breast cancer tissue (LHRH receptors stain in green and nuclei stain in blue).

The adhesion forces/interaction between the LHRH-conjugated drugs and different stages of breast tumors are presented in Fig. 8a. The results show that the pull-off forces increase with the stages of the breast tumors. The pull-off forces obtained via atomic force microscopy also revealed that the adhesion forces between the unconjugated PGS or unconjugated PTX and the breast tumors were relatively low in the early, mid and late stages of the tumors (21 ± 4.9 nN, 29 ± 2.9 nN, 26 ± 2.6 nN; and 14 ± 3.2 nN, 22 ± 4.3 nN, 34 ± 6.2 nN, respectively). In the case of LHRH-conjugated PGS and LHRH-conjugated PTX (See Fig. 8a), higher average adhesion forces were obtained for the early, mid and late stage tumors (51.1 ± 2.7 nN, 86 ± 8.6 nN, 101 ± 10 nN; and 51 ± 9.9 nN, 72 ± 3.6 nN, 81 ± 14 nN, respectively). The immunofluorescence staining (Fig. 8b–d) also revealed that the densities of LHRH receptors increased from the early, the mid and late stages of the breast tumors.

The above results suggest that the highest therapeutic activity was associated with the LHRH-conjugated PGS or LHRH-conjugated PGS PTX (Fig. 7a–c). The improved therapeutic effects of the LHRH-conjugated drugs are also associated with the increased adhesion of LHRH-conjugated drugs to the LHRH-receptors that were overexpressed on the surfaces of breast cancer cells/tissues. The presence of these receptors creates binding sites for the specific targeting of TNBC (Fig. 8b–d).

In general, the average adhesion forces between the LHRH-conjugated PGS (51.1 ± 2.7 nN) and the early stage breast tumor were nearly three times that of the unconjugated PGS (21 ± 4.9 nN). In mid stage breast tumors, the average adhesion force between LHRH-conjugated PGS and the mid-stage tumor (86 ± 8.6 nN) is about three times than that for PGS alone (29 ± 2.9 nN), while for the late stage tumor (Fig. 8a), the adhesion force of LHRH-conjugated PGS to the tumor (101 ± 10 nN) was four-fold the value to the PGS drug (26 ± 2.6 nN). This trend is similar to that obtained for LHRH-conjugated PTX and unconjugated PTX.

Thus, the increase in adhesion force is attributed to increased incidence of LHRH receptors on the surfaces of the breast tumors. The increase in the adhesion forces of the LHRH-conjugated drugs to breast tumors has been shown from prior molecular dynamic simulations^38^ to be attributed to the effects of hydrogen bonding and Van der Waals interaction between the LHRH-conjugated drugs and the TNBC tissue/cells receptors. Despite the increased in adhesion between receptors and conjugated drugs, it is very crucial to note that the conjugation of small molecules to GnRH (LHRH) may affect/block their permeability through the membrane. This process of internalization might result in changes in pH and vesicle localization (not equal to cytoplasmic release and the interaction between the small drug and intracellular tissue)^45,46^.

### Toxicity and histopathology results

**Figure 9:**
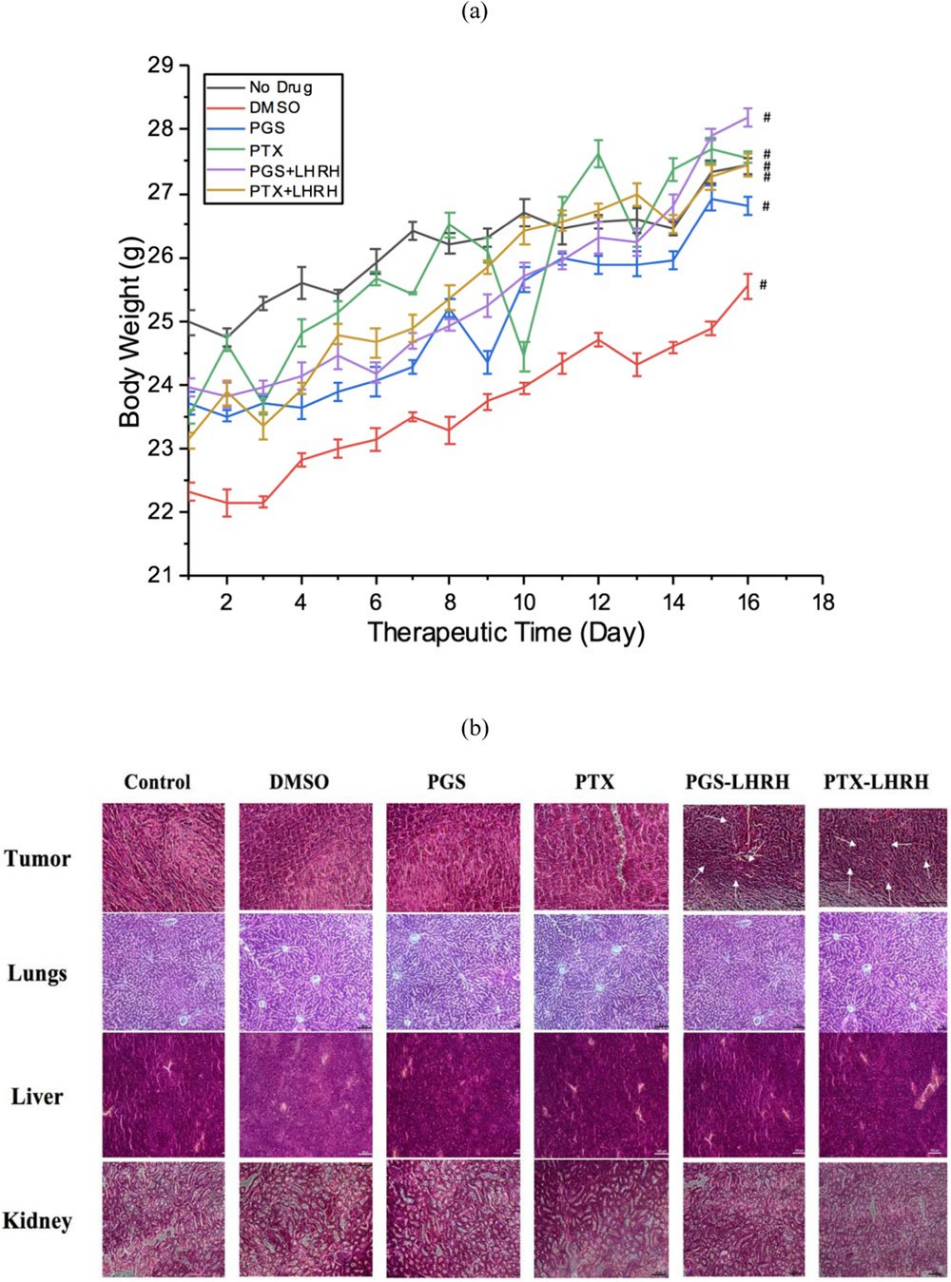
(**a**) Change in the body weight of xenograft tumor-bearing mice treated with 10 mg/kg of conjugated and unconjugated PGS and PTX drug in the presence of control (n = 3, ^#^p < 0.005). (**b**) Histopathological examination of tumor tissues and organs in MDA-MB 231 induced xenograft breast tumor model mice after treatment (from 21-day treatment group) with unconjugated and LHRH-conjugated PGS and PTX drugs.

The body weights of the tumor-bearing mice associated with the therapeutic period are presented in Fig. 9a. There were no significant changes in body weight associated with any of the dosing groups tested. Furthermore, there were no observable physiological changes, changes in mortality, or changes in the body weight after the administration of the drugs, compared to the control mice. Also, the body weights measured during the therapeutic period clearly correspond to the body weight ranges of same aged normal mice in all of the tested groups, including the control mice. Again, all of the mice appeared to be healthy with normal eyes, fur and skin conditions, during the entire study.

Histopathological examinations of tumor tissues revealed that tumor cells from the PGS-LHRH or PTX-LHRH treated mice exhibited disorder and varying cell size. They also appeared to be more mitotic. The optical microscopy images presented in Fig. 9b show the structure of the tumor tissue extracted from the xenograft breast models after treatment with LHRH-conjugated and unconjugated drugs. The stained images revealed evidence of increased angiogenesis as a result of fibrous necrosis in the tumor tissue^47^.

Treatment with LHRH-conjugated PGS/PTX resulted in higher levels of necrosis in the tumors when compared to those treated with the unconjugated PGS or PTX drugs (Fig. 9b). There were no significant histological or pathological changes in the liver, lungs, and kidneys of the mice that were treated with LHRH-conjugated PGS or LHRH-conjugated PTX or unconjugated PGS or PTX (See Fig. 9b). Hence, the features observed in the organs of targeted drug treated mice organs were comparable to those of the control mice organs.

In mice treated with LHRH-conjugated PTX or LHRH-conjugated PGS, there was no evidence of liver cell hyaline degeneration and necrosis, and no pulmonary edema or hyperplasia. There was also no evidence of renal hyperplasia, and the glomerular volume of the kidneys was normal. Furthermore, no chemotherapeutic drug-induced histological changes and tumor metastasis were observed in the LHRH-conjugated PTX or LHRH-conjugated PGS treated mice. Hence, the observed elimination and shrinkage of tumors (associated with effective targeting of the breast xenograft tumors by the PGS-LHRH and PTX-LHRH drugs) did not induce any degeneration in the primary organs.

**Figure 10:**
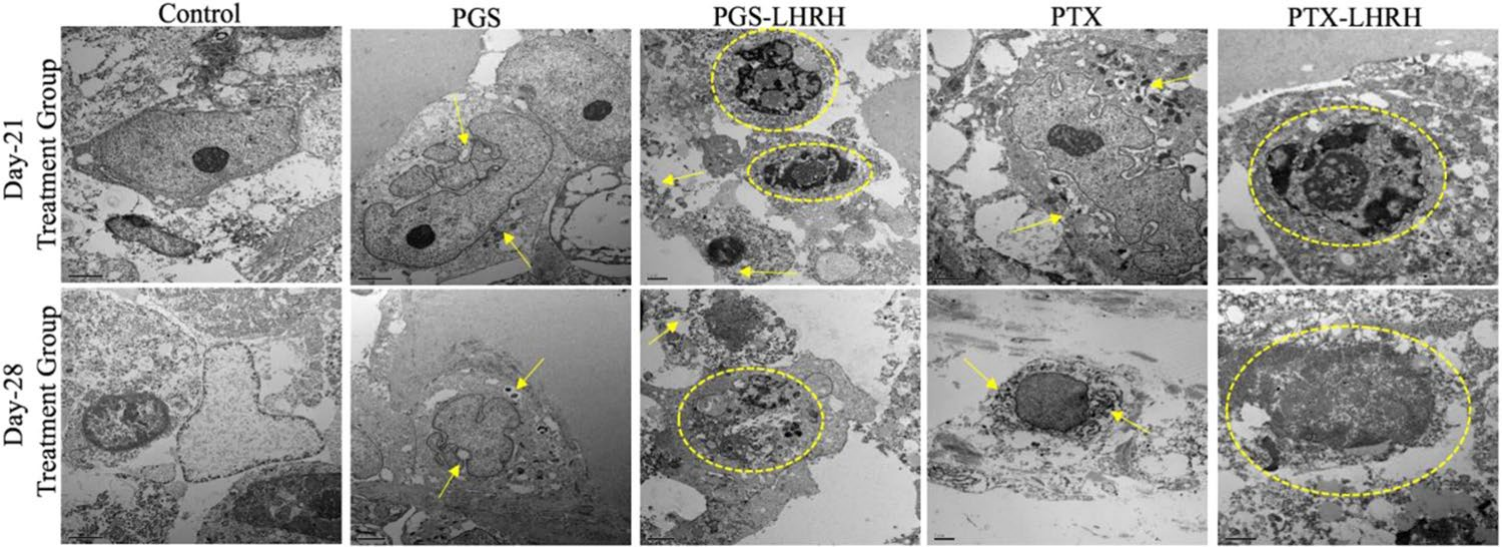
Representative TEM micrographs showing the morphologies and ultrastructures of tumor tissue/cells from MDA-MB 231 induced xenograft breast tumor model mice after treatment with PGS, PGS-LHRH, PTX, PTX-LHRH (circled and pointed structures are fragmentation of the membranes and nuclei).

Figure 10 presents TEM images of the drug treated tumors obtained from the 21-day and 28-day treatment groups. The TEM images revealed evidence of greater structural changes in the cancer cells/tissues injected with PGS-LHRH or PTX-LHRH than in those injected with PGS or PTX. The circled and pointed structures observed are changes in the structure of the membranes and nuclei are attributed to the effects of the drugs on the tumor tissue. The structural changes in the breast cancer tissues are attributed to due to drug effects on the breast cancer tissues. These include shrinkage and the disorganization of the nuclei (nuclear fragmentation) and the cell membranes that are revealed in the images of the breast cancer tissues that were obtained from animals that were treated with the conjugated drugs (PGSLHRH, PTXLHRH).

In contrast, the structure of the untreated tumor tissue did not reveal significant changes in the tissue structure in control experiments that were carried out without the injection of targeted drugs (PGS-LHRH and PTX-LHRH). The current results, therefore, suggest that the LHRH-conjugated drugs induced more significant changes in the structure and morphology of the breast cancer cells and tissue.

### Implications

The implications of this work are significant for the design of targeted drugs for the specific and localized treatment of TNBC. First, we have shown that the conjugation of LHRH peptides to cancer drugs (such as PGS and PTX) significantly enhance the delivery of such drugs to TNBC tumor sites. The specific delivery reduces the side effects associated with bulk chemotherapy. The corresponding increase in adhesion forces (between LHRH-conjugated drugs and the breast tumors) is also attributed to the increase in hydrogen bonds and Van der Waal’s interactions between LHRH (attached to the drug) and the overexpressed LHRH receptors on the surfaces of the breast cancer cells/tissue^48^. Although, the expressed LHRH receptors shown via immunofluorescence staining may quantitatively support the claims on the adhesion results that the LHRH ligands are binding to the receptors expressed on the cells/tissue. However, the results from our siRNA knock down, RT-qPCR quantification and alamar blue cell viability studies show evidence that the receptors help to promote specificity of the conjugated drug to the LHRH receptors of the MDA-MB-231 cell lines considered. Furthermore, there is a need to gain more insights and unravel direct evidence of interactions or binding between LHRH ligand and their receptors expressed on cells/tissues using a combined knowledge of tagged fluorescence receptors and CRISPR analysis.

Also, in the case of the xenograft tumors that were induced subcutaneously at the interscapular sites, the intravenous injection of LHRH-conjugated prodigiosin via the tail vein, resulted to elimination or shrinkages of the induced tumors at the different stages (early, mid and late stages) of tumor development (Fig. 7a–c). Furthermore, the LHRH conjugation of the prodigiosin and paclitaxel also significantly enhanced the specific targeting of TNBC in the athymic nude mouse model that was used in the current study. The side effects associated with the specific delivery of the drugs were also minimal.

It is important to note that the injection of 10 mg/kg of LHRH-conjugated prodigiosin or LHRH-conjugated paclitaxel eliminated the tumors that were formed within the early stages of tumor development (within 14 days). This tumor elimination was achieved without any evidence of toxicity (Figs. 7a and 9). The same concentration of drug also resulted in significant shrinkage of the mid- and late-stage tumors that were formed after 21 and 28 days, without any noticeable toxicity (Figs. 7b,c and 9). This suggests that extended treatments (beyond the two-week injection period that was explored in this study) could result in the possible elimination of mid-and late- stage tumors.

Hence, the results obtained from the *in vitro* cell viability study, immunofluorescence staining, drug-tumor adhesion measurements and *ex vivo* histopathology/microscopy revealed that the improved therapeutic effects are associated with the increased adhesion forces/interactions of the LHRH-conjugated cancer drugs (PGS-LHRH and PTX-LHRH) to LHRH receptors that are overexpressed on the surfaces of triple negative breast cancer cells/tumors. These give rise to improvements in the specific targeting of TNBC cells/tissue and the elimination or shrinkage of TNBCs that were observed in this study. In future, additional tests are needed to demonstrate the observed effects of the conjugated and unconjugated drugs on cohorts of at least 6 animals per group to validate this pioneering work under *in vitro* conditions. Further work is needed to study the effects of different drugs concentrations in animal models to target different types of cancer (like pituitary cells) that expresses LHRH receptors.

## Conclusion

We have successfully developed specific LHRH-conjugated PGS drug that target overexpressed LHRH receptors on TNBC cells/tissue under *in vitro* and *in vivo* conditions. Our results suggest that the specific targeting is enhanced by increased adhesion of the LHRH-conjugated drugs to TNBC cells/tissues under *in vitro* and *in vivo* studies. The LHRH-conjugated drugs (PGS-LHRH and PTX-LHRH) also increase the inhibition of MDA-MB-231 TNBCs more than the unconjugated drugs (PGS and PTX).

The results obtained from our siRNA knockdown experiments showed that siRNA 1 and siRNA 2 knocked down LHRHR transcripts by 70% and 90%, respectively. The results from our knocked down receptor/cell viability studies demonstrate that LHRH receptors on the MD-MB-231 cells can mediate ligand-conjugated drug entry into the cells to increase cell death. In the case of the *in vivo* studies, our results show that the double injection of 10 mg/kg of LHRH-conjugated prodigiosin or LHRH-conjugated paclitaxel (within a two-week period) eliminates early stage breast tumors (14-days tumor-treatment group). The results also show that similar dosage administered significantly shrunk mid (21-days tumor-treatment group) and late stages (28-days tumor-treatment group) tumors in the athymic nude mice.

Also, the adhesion forces/interactions between the LHRH-conjugated drugs (PGS or PTX) and the breast cancer tissue were about 3–4 times those between the unconjugated drugs (PGS or PTX) and TNBC tissue. Hence, the increased specificity of the LHRH-conjugated drugs (in the targeting of TNBC) is attributed to the increased adhesion of the LHRH to the LHRH receptors that are overexpressed on the surfaces of the TNBC cells/tissue during the early, mid and late stages of TNBC progression.

Furthermore, the *ex vivo* histopathological results revealed no evidence of physiological changes due to LHRH-conjugated drug administration. No adverse differences in mortality, or changes in body weight were observed, compared to control mice, after treatment with PGS-LHRH or PTX-LHRH drugs. This suggests that the proliferation of the induced xenografts TNBC tumors in the athymic nude mice was specifically and robustly inhibited by PGS-LHRH or PTX-LHRH. Hence, the current results show that LHRH-conjugated PGS and LHRH-conjugated PTX significantly enhance the specific targeting and localized treatment of TNBCs without adverse toxicity effects.

## Experimental Section

### Materials

Prodigiosin was biosynthesized from *Serratia marcescens*^35,36^, while paclitaxel, (N-hydroxysuccinimide (NHS), 1-ethyl-3-(3-dimethylaminopropyl)carbodiimide hydrochloride (EDC HCl), Alamar Blue Assay (ABA) kits, Dubecco Phospate Buffer (DPBS), 12-well plate, and opaque 96-well plates were purchased from Thermo Fisher Scientific (Waltham, MA, USA). N,N-Dimethylformamide (DMF), 2-Ethoxy-1-ethoxycarbonyl-1,2-dihydroquinoline (EEDQ), Dimethyl sulfoxide (DMSO), [D-Lys6]LHRH peptide, anhydrous pyridine, succinic anhydride and silica were all obtained from Sigma-Aldrich Co. LLC, (St. Louis, MO USA). Also, 3 kDa Amicon Ultra -4 Centrifugal Filters Units and Amicon Pro Purification System were purchased from Millipore Sigma (Burlington, MA, USA). The growth media (L15), fetal bovine serum (FBS), and the human triple negative breast cancer cell line (MDA-MB-231) that was used for *in vitro* cytotoxicity/cell viability study and to induced subcutaneous tumors, were all purchased from American Type Culture Collection (ATCC, Manassas, VA, USA).

Finally, penicillin/streptomycin, a cell medium supplement and antibiotic was obtained from Thermo Fisher Scientific, Inc. (Waltham, MA, USA). Athymic Nude-Foxn1nu strain mice with individual weights of ~17 g was purchased from Envigo (South Easton, MA, USA). All of the animal protocols were approved by the Institutional Animal Care and Use Committee (IACUC) at the University of Massachusetts Medical School, Worcester, MA (IACUC docket # A2630-17) and carried out in accordance with the guidelines published by the National Institute of Health Guide for the Care and Use of Laboratory Animals.

### Experimental procedure

#### Prodigiosin synthesis and conjugation

Prodigiosin (PGS) was synthesized in the Soboyejo Research Group at WPI, as reported in our previous work^35,36^. A PGS concentration of 31.15 mg/ml was prepared with DMSO. The resulting solution of PGS was activated by adding 5.14 mg/ml of NHS under gentle agitation at room temperature (23 °C) for 2 h. The EDC HCl linker was then added to the PGS-NHS solution to form PGS esters. This was done in a dark enclosed area for 18 h at a temperature of 4 °C. Supernatant formed from the mixture was separated from a precipitate by centrifugation at 4500 rpm for 15 mins. The centrifugation was done using an Eppendorf 5804 Benchtop Centrifuge with an A-4-44 Rotor (Eppendorf, Hauppauge, NY, USA). The resulting supernatant was then concentrated under vacuum for 24 h before running it through a silica-loaded gel column chromatography system (Corning LG4564T-104 Glass Chromatography, Cole-Parmer, Vernon Hills, IL) to purify the solution.

After activation and binding with the linker, as described above, the resulting solution of active derivatives of PGS was then incubated with 1 mg/ml of [D-Lys6]LHRH. This was done to chemically conjugate PGS to LHRH. The conjugation process was carried out by swirling at 600 rpm in a test tube within a dark enclosure for 18 h at 4 °C. Excess and unconjugated LHRH, as well as the excess derivative of PGS, were removed from the conjugated drug molecules using 3 kDa Amicon Ultra -4 Centrifugal Filters Units, followed by an Amicon Pro Purification System (Millipore Sigma, Burlington, MA, USA). The conjugation of LHRH to PGS was then confirmed with FTIR, while the purity was analyzed using LC-MS.

#### Paclitaxel conjugation

The paclitaxel (PTX) # P3456 that was used in this study was purchased from Thermo Fisher Scientific (Waltham, MA, USA). It was activated with 2-hydroxyl groups. Since the coupling of PTX directly to LHRH peptides was not favorable, a two-step coupling process was used to couple LHRH to PTX. First, esters of PTX were formed by modifying a method reported by Deutsch *et al*.^49^, to form 2′-O-paclitaxel succinate (a hemisuccinate). The esters were formed by using PTX and succinic anhydride. These were dried for 24 h in the presence of silica gel that was fused with calcium chloride at room temperature (~23 °C) in a high-vacuum desiccator.

The dried PTX was dissolved in anhydrous pyridine, followed by the addition of a solid form of succinic anhydride. The combined solution was then stored at room-temperature (~23 °C) under argon gas in a 3-neck sealed flask for 12 h to form 2′-O-paclitaxel succinate (PTXSCT). Subsequently, silica gel was used to purify the resulting solution via column chromatography, with chloroform as a solvent (for column packing and product loading)^38^.

The conjugation of PTXSCT to [D-Lys6]LHRH involved the initial activation of PTXSCT with freshly prepared NHS and EEDQ linker in dry DMF, and gentle stirring at 4 °C for 3 h. The resulting solution (containing DMF solution of the PTXSCT activated ester) was then added to the [D-Lys6]LHRH and gently vortexed at 600 rpm for 6 hours at 4 °C to form LHRH-conjugated paclitaxel drug. The conjugated drug molecule was separated using a combination of 3 kDa Amicon Ultra-4 Centrifugal Filters Units, and a Amicon Pro Purification System. The conjugation was confirmed with FTIR, and further characterized with LC-MS.

#### Drug characterization (FTIR and LC-MS)

PGS, PTX and their conjugated components (PGS-LHRH and PTX-LHRH) were analyzed using Attenuated Total Reflectance Fourier Transform Infrared spectroscopy (ATR-FTIR) (IRSpirit, Shimadzu, Kyoto, Japan). The FTIR was set to absorbance mode in an effort to investigate the functional groups, bonding types, and the chemical characteristics of the novel compounds.

Furthermore, an Agilent 1200 LC/MS system equipped with a 6130 series (Santa Clara, CA, USA) single-quadrupole was used to analyze the purity of the conjugated drugs. The drug samples were ionized using an electrospray source with polarity switching (±ESI). The Ionized species were analyzed at an m/z range between 180 and 1200. This was done using the gradient method under acidic conditions.

The mobile phase components were A1: 95% H_2_O 5% acetonitrile containing 0.1% formic acid, B1: 5% H_2_O 95% acetonitrile containing 0.1% formic acid. These were identified with a diode array detector that simultaneously monitors the following three UV wavelengths: 210 nm, 254 nm, and 277 nm. In each LC-MS test, 2 µl of sample was injected. Mobile Phase Composition: 5%B for 0.5 min., 8 min. gradient to 100%B, hold 1 min., 0.5 min. gradient to 5%B, hold 4 min. The total data acquisition time was about 18 minutes per sample.

#### LHRH Receptors Staining, siRNA knockdown, RT-qPCR quantification, Cancer Cell Viability and Drug Uptake Studies

Immunofluorescence staining of the LHRH receptors on the cells were carried out as reported by prior work^20^. siRNA knockdown and RT-qPCR quantification of LHRH receptor were carried out to explore drug-ligand receptor interaction. Predesigned DsiRNAs (hs.Ri.GNRHR.13) against LHRH receptor (GnRHR) were obtained from IDT (Coralville, Iowa, USA). 1 × 10^5^ MDA-MB-231 cells were seeded per well in 24-well plate. After 24 hours, the cultured cells were transfected with 10 nM DsiRNAs using Invitrogen Lipofectamine 3000 Transfection Reagent (ThermoScientific, Waltham, MA, USA) according to the manufacturer’s instruction. 48 hours after transfection, cells were harvested and total RNA was isolated and cDNA was synthesized using SV Total RNA Isolation and GoScript Reverse Transcription kits from Promega (Madison, WI, USA). Primers for RT-qPCR were purchased from Genewiz (Cambridge, MA, USA) and RT-qPCR was performed using PowerUp SYBR Green Master Mix (Applied Biosystems, Foster City, CA, USA). Reactions were run and analyzed on QuantStudio 5 Real-Time PCR machine, and results were normalized against GAPDH expression.

Alamar Blue (AB) cell viability and cytotoxicity assay was used to study the MDA-MB-231 cells lines in the log phase of growth under *in vitro* conditions. First, MDA-MB-231 cells were harvested with trypsin-EDTA in the presence of Dulbecco Phosphate Buffer (DPBS). 10^4^ cells/well were seeded in 12-well plates with L15+ medium (L15 medium with cell medium supplement of FBS and penicillin/streptomycin). After a 3-hour attachment period (of the cells), a concentration of 5 µM of LHRH, prodigiosin, paclitaxel, LHRH-conjugated prodigiosin, LHRH-conjugated paclitaxel and DMSO (in culture medium) were added to each well of the 12-well plates consisting of 10^4^ cells. Effect of concentration was further explored with concentrations of 15 µM, 25 µM and 30 µM, respectively. The se concentrations are within the recommended range^16,50^.

Cell viability and cytotoxicity were monitored using the alamar blue cell viability and cytotoxicity reagent (Thermo Fisher Scientific, Waltham, MA, USA) during incubation times of 0 h, 18 h, 24 h, 48 h and 72 h, with the drugs at 37 °C. At each time point, the culture medium was replaced with 1 ml of 10% AB solution (in the culture medium). After each time point, 100 µl of the solution incubated with alamar blue solution (ABS) was transferred into triplicate wells of black opaque 96-well plate (Thermo Fisher Scientific, Waltham, MA, USA).

The fluorescence intensities of the cell medium supernatant incubated with ABS were measured at 544 nm excitation and 590 nm emission using a 1420 Victor3 multi-label plate reader (Perkin Elmer, Waltham, MA, USA). The percentage of alamar blue reduction (the percentage difference in cell population between the treated and untreated cells) and the percentage of cell growth inhibition were determined using a combination of the ABS and cell viability studies. In this way, the cytotoxicity of the respective conjugated drug molecules was obtained from Eqs. 3 and 4 below. These (% Reduction and % Growth inhibition) are given by:

3 $$ \% \,Reduction=\frac{F{I}_{sample}-F{I}_{10 \% AB}}{F{I}_{100 \% R}-F{I}_{10 \% AB}}\times 100$$

4 $$ \% \,Growth\,Inhibition=\left(1-\frac{F{I}_{treated}}{F{I}_{untreated}}\right)\times 100$$

where FI_sample_ is the fluorescence intensity of the (treated or untreated) cells, FI_10%AB_ is the fluorescence intensity of 10% AB reagent (negative control), FI_10%R_ is the fluorescence intensity of 100% reduced alamar blue (positive control). FI_treated_ is the fluorescence intensity of treated cells, and FI_untreated_ is the fluorescence intensity of the untreated cells.

Finally, *in vitro* cell fluorescence staining (of drug-interacted cells) was used to study the effects PGS, PGS-LHRH, PTX and PTX-LHRH drugs on the actin and vinculin cytoskeletal structures in of MDA-MB-231 breast cancer cells. First, breast cancer cells were incubated for 6 hours with 30 µM of each drug. The incubated cells were then washed with PBS and fixed with 4% paraformaldehyde for 15 minutes. The resulting cells were permeabilized by incubating with 0.1% Triton X-100 for 15 minutes followed by blocking with 1% BSA for 1 hour at room-temperature (25 °C). The samples were then further stained with primary antibody Vinculin Mouse Monoclonal Antibody (Product # MA5-11690) and incubated for 3 hours at room-temperature. This was followed by labeling with a secondary antibody [Goat anti-Mouse IgG (H+L) Superclonal™, Alexa Fluor® 488 conjugate (Product # A28175)] for 45 minutes at room-temperature. The actin cytoskeleton was then stained with Alexa Fluor 555 Rhodamine Phalloidin (Product # R415, 1:300, Thermo Fisher Scientific, Waltham, MA, USA).

Finally, the nuclei were counterstained with SlowFade Gold Antifade Mountant with DAPI (Product # S36938, Thermo Fisher Scientific, Waltham, MA, USA). The resulting stained samples were then imaged with a Leica SP5 Point Scanning Confocal Microscope (Leica TCS SP5 Spectral Confocal couple with Inverted Leica DMI 6000 CS fluorescence microscope, Leica, Buffalo Grove, IL, USA) equipped with a 60X magnifying lens.

#### *In vivo* Tumor Development and Targeted Drug Delivery

In this section, cell culture, tumor induction, and drug injection studies were conducted. First, 20 µl of 1 × 10^6^ MDA-MB-231 human triple negative cancer cells were cultured in T75 tissue culture flasks (CELLTREAT, Pepperell, MA, USA). This was done at 37 °C until 70% confluence was reached. The cells were grown under normal atmospheric pressure levels in an “L15^+^ medium” that consisted of: L-15 medium (ATCC, Manassas, VA, USA) supplemented with 100 I.U./ml penicillin/100 lg/ml streptomycin and 10% FBS (ATCC, Manassas, VA, USA).

Forty 4-week-old female Athymic Nude-Foxn1nu strain mice of ~17 g each were purchased from Envigo (South Easton, MA, USA). These animals were approved for use in the current work by the University of Massachusetts Medical School Institutional Animal Care and Use Committee (UMMS IACUC). All of the animals were maintained in accordance with the approved UMMS IACUC procedures and guidelines.

Subcutaneous tumor xenografts were induced by the injection of 5.0 × 10^6^ of MDA-MB-231 human triple negative breast cancer cells (suspended in sterile saline) into the interscapular region (for a better angiogenic response) of each of the mouse. Tumor formation was carefully assessed by palpation and MRI. Tumor growth was then monitored daily with the digital calipers. The tumor volume was calculated using the following modified ellipsoidal formula^51,52^:

5 $${\rm{Tumor}}\,{\rm{Volume}}\,({\rm{TV}})=\frac{Widt{h}^{2}\times Length}{2}$$

where the *Length* was the longest axis of the tumor and the *Width* was the longest measurement at a right angle to the length.

The mice were randomly assigned into treatment groups of three (for each drug injection) due to the pioneering studies as well as the animal welfare. These included groups of mice designated for early stage tumors (14 days after tumor induction), mid stage tumors (21 days after tumor induction), and late stage tumors (28 days after tumor induction) tumors. The weights of the mice and their tumor sizes were monitored and measured daily using digital calipers. The instantaneous tumor volumes and body weights were used to guide the subsequent administration of the drugs. They were also used to monitor toxicity and side effects associated with the drugs administered. For each of the study groups, 3 mice were randomly assigned to injections of 10 mg/kg of the specific drug configuration (PGS, PTX, LHRH-conjugated PGS, LHRH-conjugated PTX and DMSO).

The different groups of mice were injected intravenously with each drug through their tail veins. This was done after tumor growth for 14, 21 and day 28 days. The mice were injected with 10 mg/kg per week, during the two-week periods in which the effects of drugs were investigated under *in vivo* conditions. Following each drug administration, the tumor sizes were monitored with calipers on a daily basis (every 24 hours). This was done to monitor possible tumor shrinkage, growth or elimination. At the end of the treatment protocol for each treatment group, the mice were euthanized. The tumor tissues were then excised from all of the mice, including tissues from their major organs (kidneys, liver and lungs).

#### Immunofluorescence Staining of Overexpressed Receptors

Immunofluorescence (IF) staining was used to characterize the overexpressed receptors on the triple negative breast cancer tumor using the method described in prior work^20^. The IF staining was used to study the distributions of LHRH receptors that were over-expressed on the breast tumors. Tissue from the frozen tumors were embedded slowly in optimum cutting temperature (OCT) compound. This was done in a cryostat (Leica CM3050 S Research Cryostat, Leica Biosystems Inc., Buffalo Grove, IL, USA) that was used to ensure that the tissues did not thaw. 10 µm thick slices were obtained from specific frozen breast cancer tumors (obtained from the nude mice) that were then sectioned on charged glass slides using a Leica cryomicrotome (Leica Biosystems Inc., Buffalo Grove, IL, USA).

The sliced sections were allowed to dry overnight at room-temperature (~23 °C). This was done to facilitate adhesion to the glass slides for subsequent immunofluorescence staining. Following their adherence to glass slides, the sliced tumor samples were incubated with 0.5 ml of 3% bovine serum albumin (Sigma-Aldrich, St. Louis, MO, USA) (blocking agent) prepared with PBS mixed with 30 µl of triton X-100 (Life technologies Corporation, Carlsbad CA). This was done at room-temperature (~23 °C) for 60 mins.

The blocking agents were aspirated from the samples, and then incubated with drops of 100 µl of anti-LHRH Antibody (Millipore Sigma, Burlington, MA, USA) a primary antibody, to detect the levels of LHRH. This was done using a concentration of 1 µg/ml in a desired dilution. The resulting samples were then incubated overnight at 4 °C before dip-rinsing three times (1 min each) in 1X PBS. The treated tumors were further incubated with 50 µl of anti-mouse IgG conjugated with Alexa fluoro 488 secondary antibody with concentration of 1 µg/mL for 2 hours. This secondary antibody was prepared at a concentration of 1 µg/ml in 1% BSA solution. Both the primary and secondary antibody kits were purchased from Thermo Fisher Scientific, Inc. (Waltham, MA, USA). The stained samples were then rinsed thrice in 10 ml 1X PBS for 1 min each.

Finally, the cell nuclei of the tumor samples were stained with drops of 5 µg/ml of ProLong Gold antifade reagent with DAPI (Thermo Fisher Scientific Inc., Waltham, MA, USA). The resulting samples (on the glass slides) were fixed with coverslips using a few drops Permount Mounting Medium. The stained samples were then imaged at a magnification of 60x with Leica SP5 Point Scanning Confocal Microscope (Leica TCS SP5 Spectral Confocal couple with Inverted Leica DMI 6000 CS fluorescence microscope, Leica, Buffalo Grove, IL, USA).

#### Drug-Tissue Adhesion Study

In an effort to understand the specificity in the targeting of triple negative breast cancer via the receptors that are over-expressed on the tumor using the LHRH-conjugated drugs, *ex vivo* adhesion measurements at nanoscale with Atomic Force Microscope (AFM) (Asylum Research, Oxford Instrument, CA, USA) were carried out on the control xenograft tissue samples at different stages of tumor development. Adhesion forces and interactions (between the different drug molecules and receptors on the surfaces of the tumor tissues at different stages of development) were measured in an effort to understand the interactions of the drugs with the tumors.

Adhesion measurements were carried out on the 10 µm thick microtome tissue slices. These sliced tissues were used for adhesion measurements in an Asylum MFP3D-Bio AFM. The RESP-20 AFM tips AFM tips (Bruker Santa Barbara, CA, USA) were dip-coated with prodigiosin or paclitaxel or LHRH-conjugated prodigiosin or LHRH-conjugated prodigiosin using the techniques described in ref. ^20^.

A simple AFM tip dip-coating technique^20,28,36^ (of the drugs) was used to coat the AFM tips at room-temperature (~23 °C). In addition, a positive control of LHRH peptides was coated onto some AFM tips and used to determine the adhesion forces between the coated AFM tips and the receptors on the surfaces of the breast cancer tissue slices. All of the coated AFM tips were air-dried for about 6 h and kept overnight in a desiccator before the adhesion measurements.

The spring constants of the coated and uncoated AFM tips were measured experimentally using the thermal tune method^20^. This was done to obtain the actual spring constants that were used to calculate the pull-off forces from Hooke’s law. The adhesion interactions were measured for the following configurations of drug coatings on the AFM tips and breast cancer tumor tissue at different stages: bare AFM tip to breast cancer tumor; LHRH-coated AFM tip to breast cancer tumor; LHRH-prodigiosin coated AFM tip to breast cancer tumor; LHRH-paclitaxel coated AFM tip to breast cancer tumor; prodigiosin-coated AFM tip to breast cancer tumor and paclitaxel-coated AFM tip to breast cancer tumor.

#### Toxicity, Histopathological and Electron Microscopy Studies

Following the two doses of 10 mg/kg of PGS, PTX, PGS-LHRH and PTX-LHRH that were administered (on a weekly basis for two weeks) to the athymic female nude mice (subcutaneously-induced with TNBC) for tumor shrinkage/treatment, qualitative toxicity was characterized by accessing differences in mortality, changes in body weight, of poor health, general observations, and the histopathology of the lungs, kidneys and the liver at different stages of tumor development. Daily observations and weight measurements were also used to check for potential adverse reactions to the drugs, physiological changes, weight loss/gain, and the general well-being of the mice for the different treatment groups.

Tissues samples (from the 21-day treatment group) extracted from the kidneys, lungs, liver and tumor regions of the mice at the end of each study were fixed immediately in 4% paraformaldehyde, dehydrated in a graded series of alcohol, and embedded in paraffin. Hematoxylin and eosin (H and E) staining was also carried out to identify tumor necrosis and examine histologic changes in vital organs following the administration of the drugs. Briefly, formalin-fixed, paraffin-embedded tissue/organs (tumor, kidneys, liver and lungs) samples (5 μm) for mice that were injected with PGS, PTX, LHRH-conjugated PGS, LHRH-conjugated PTX drugs and DMSO. These were hydrated by passing them through decreasing concentrations (100, 90 and 70%) of alcohol baths and water. The hydrated tissue sections were then stained in hematoxylin solution for 5 mins. This was followed by rinsing with tap water for 3 minutes and differentiation in 1% acid alcohol for 5 minutes. Tap water was then used to rinse (three times), before dipping the sections in ammonia water for 2 minutes. This was followed by staining with eosin for 10 mins. The treated sliced samples were dehydrated in solution with increasing concentrations of alcohols, followed by xylene. Finally, a few drops of Permount Mounting Medium was used to mount the resulting samples. The stained slides were finally imaged with a 20x objective lens using a TS100F Nikon microscope (Nikon Instruments Inc., Melville, NY, USA) coupled with a DS-Fi3 C mount Nikon camera.

Tumors extracted from the different mice at the mid-stage tumor (day-21) and late (day-28) stages were fixed immediately in 2.5% glutaraldehyde in 0.1 M Sodium Cacodylate buffer pH 7.2, along with their respective control tumors that were not subjected to drug treatment. Prior to the processing of the fixed tumors, the tumors were transferred into freshly prepared 2.5% glutaraldehyde in a 0.1 M Sodium cacodylate buffer, and left overnight at 4 °C. The samples were then rinsed thrice in the same fixation buffer and post-fixed with 1% osmium tetroxide for 1 h, at room-temperature (~25 °C). The samples were then washed twice with DH_2_O for 10 minutes, before dehydrating through a graded series of 60%, 80% and 90% ethanol. This was followed by three changes in 100% ethanol.

The samples were then infiltrated, first with two changes of 100% propylene oxide, and finally with a 50%/50% propylene oxide/SPI-Pon 812 resin mixture. They were then left overnight in the resin mixture. On the next day, the samples were put through five changes of fresh 100% SPI-Pon 812 resin. They were then polymerized in the embedding molds for 24 hours at 68 °C. Ultra-thin sections with thicknesses of ~ 70 nm were obtained using a diamond knife (Diatome, Hatfield, PA) that was mounted in the Ultramicrotome Leica EM UC7 system (Leica Biosystems Inc., Buffalo Grove, IL, USA). These thin sections were placed on copper support grids and contrasted with lead citrate and uranyl acetate for transmission electron microscopy. Subsequently, the ultrathin sections were examined in a CM10 TEM (FEI Technologies Inc., Hillsboro, Oregon, USA) that was operated at an accelerated voltage of 80 kV. The TEM images were captured using a Gatan CCD camera (Ultrascan 4000 CCD, Pleasanton, CA, USA).

### Statistical analysis

An OriginPro 2017 software package was used to analyzed the statistical data. Independent Student t tests and one-way analyses of variance (ANOVA) were used to study the differences between the control and the study groups. The statistical significance in the survival (%) of drug treated mice versus the control mice were statistically evaluated by using the differences in their population means. In this way, the effects of the cancer drugs (PGS, PTX, PGS-LHRH and PTX-LHRH) were evaluated statistically. A p-value < 0.05 was considered to be significant.
